# Homœopathy and Surgery in Their Relation to Gynecology

**Published:** 1896-09

**Authors:** Eric Vondergoltz

**Affiliations:** Professor of Gynecology


### HOMŒOPATHY AND SURGERY IN THEIR RELATION TO GYNECOLOGY.

Eric Vondergoltz, M. D., Professor of Gynecology.

(A lecture before the Summer Class '96, delivered on special request at the Metropolitan Post-Graduate School of Medicine, at New York City, Saturday, July 18th, 1896.)

Ladies and Gentlemen:—By your special request I will here give a picture of the treatment of gynecological cases regarding pure medical dosage and the dividing of clinical material between pure medical therapeutics and surgery.

Let me speak first of the medical treatment; the selection of the homœopathic drug in my clinic here, is in conformity with the method of von Bœnninghausen's *Pocket-Book* edited by Dr. T. F. Allen.

As you all are familiar with the demands of the materia medica regarding symptoms, localities, and modalities, you will consent to the fact that the selection of a remedy covering all symptoms, or at least the greater portion, often will prove extremely difficult.

The want of a helping index for such a symptomatological dilemma—already felt in the first decades of Homœopathy—was successfully overcome by von Bœnninghausen, the most brilliant pupil of Hahnemann.

When I began Homœopathy I learned the key-note system, but by no means was I satisfied, as the plurality of failures caused me to wish for a better and more reliable system.

Finally I became acquainted with von Bœnninghausen's system, which means that all localities, all symptoms, and all modalities shall be registered according to the numeral qualities as given by the author of the *Pocket-Book.*

In many cases I met with unforeseen results, but very often this system also failed.

As I saw that no other system besides the two just mentioned existed, and thinking that in von Bœnninghausen's system, now before the homœopathic profession for nearly one hundred years, the tedious working of the cases was not forgotten and pushed to the wall, I began to try to find the reason how these failures of mine could happen.

More than in any other branch of medicine the gynecologist must deal with reflex ailments, for example I had to deal with paralytic face-atrophy, throat troubles, hemiplegias of uterine origin, which quickly vanished and were cured by having struck the right remedy for the sexual zone. This caused me to single out in my gynecological cases the primary pathological seat of the disease, furthermore the principal gynecological symptoms and modalities.

With only these data I began to work out the cases, as I could notice, that if I registered facial troubles, etc., the plurality of secondary symptoms finally overwhelmed the gynecological zone in regard to quantity, and especially quality of remedies, so that, especially in one case where after having worked the gynecological case in my way, and gaining as the highest remedy Platinum, which remedy was given, and which cured the case in short time, although the found remedy after having taken in all and all symptoms and localities differed materially from Platinum, viz., Nux-vom.

The interesting facts of this case with Platinum and Nux-vom. are these—that the clinical features claim both remedies for facial neuralgias, but as we also know that Nux-vom. belongs to the class of so-called idiopathic brain and nerve-drugs, where Platinum in its most absolute prominence belongs to genital idiopathic affections with a certain predilection for the female sex.

I believe that just this observation can be given as a clinical proof for the necessity of specialists in the homœopathic school, as the specialist must select the cases belonging to the domain of his special field by proper physical examination and special diagnosis.

The foregoing case had gone through the treatment of different homœopaths of excellent standing without any beneficial result. Many a time in working after von Bœnninghausen's method, we will see that all symptoms, and especially the most necessary ones are not found in the small *Pocket-Book*, or we will come to the uncertainty of finding not only two, but six remedies of more or less equal value.

In all these cases it has proved rational and successful to add such not-mentioned symptoms from T. F. Allen's general symptom register of pure materia medica, that finally enabled me to get one remedy as the highest in value.

Besides this manner of working out von Bœnninghausen's method, I was lately forced by necessity to dispose of my enormous clinical material, to use a somewhat more simplified way. I call this manner the elective way—that is, that from other prominent secondary symptoms and localities—if sharply defined—I gather very well-known keys. This seeming paradox is explained by the fact that certain remedies correspond to certain maladies, and that herein again a certain class of symptoms will be mentioned by the patient with a nearly mathematical exactness; these circumstantial points are now the best affirmatives for the remedy in quest. For example, in one case, sent from the Throat Hospital in Twenty-third Street and Third Avenue, I could effect a cure of very complicated gynecological ailments in considering the throat. I was considering among other remedies—Causticum, and now this Causticum fully cured her parametritic exudation of the right side. This elective method is rather unreliable, and only justifiable by circumstances, as for administering in large clinics, etc., but I must warn you positively not to indulge in this method privately, where time will be at your disposal.

The record of the clinic in May and June states that the time alotted to each patient was six minutes; if now in this short time personalia, symptoms and history, physical examination, diagnosis, and finally treatment with the dictated translation to the existing physician is done, very little time can be allowed for materia medica, especially where students wish to be shown all possible phases of gynecological diagnosis.

Ladies and gentlemen, you have seen my clinics, and you have witnessed my methods; you can judge for yourselves the efficiency of the application of therapeutics in this clinic.

It is now that I must speak about the reciprocal relation between materia medica and surgery—based on Hahnemann's *Organon.*

One of the earliest and most vexing questions I encountered when turning to Homœopathy, was to differentiate between the knife and the remedy.

On one side I could see that the gynecologist, more or less, lost all sympathy with materia medica, and became a pure and simple surgeon; on the other side the homœopath, while putting aside any and every pathological macroscopic fact, lost himself in dreamland—became a faith-curer through lack of diagnosis and physical examination.

It was, therefore, for me as a beginner, not easy to remain indifferent, and to find out the trouble impartially.

And now I will describe how finally I attained a certain mode of discriminating.

Leaving all authors and all books out of this present lecture I will refer you merely to the cases at the clinic of the post-graduate.

When I took charge of one of the three gynecological departments in 1894, I was very anxious to execute my office so as not to offend the homœopathic feeling of any one by my way of treating the individual case.

Not to err in one direction nor in another, I laid down the rule to consult closely Hahnemann's *Organon*, reasoning that in reliance on this corner-stone of Homœopathy I could stand any criticism.

In every case coming under our observation we must make an inquiry in the following manner: Personal complaints, physical symptoms, prominence in either direction. By this analytic way we gain an understanding of the gravity of the disease. This finally gives us a better understanding of the anatomical facts in their influence on the organisms.

The best illustration of this will be found in the twofold treatment of prolapsus of the genitals.

Here we see the homœopaths divided in two sections, one claiming the absolute necessity of surgical interference, the other most emphatically denouncing any and every surgical procedure, recommending only the potentized drug.

Immediately the question arises—What treatment is right?

My observation in this puzzling state on the hand of the foregoing method, led me to the following result, *that both sections of homœopaths were right and both were wrong at the same time!*

The close examination will easily divide prolapsus into certain groups, which again, according to predisposing moments, either going out from the reaction of the organism in toto, or going out from a noxious local cause, which either again is constituted by a passing weakness (locally or in general) or finally is effected by a material damage.

It will be understood, therefore, that without following any further the many under-divisions of prolapsus, *the treatment can be neither absolutely medical nor absolutely surgical in toto.*

Furthermore it must be added here, that the shorter or longer time in the existence of prolapsus in its consecutive states, namely, hypertrophy, it never can and never will be judged at once as to its importance. If we are now following up prolapsus in all its movements, we will easily find *when to operate and when not;* therefore we see so often cystocele and rectocele reappearing, not as a proof of the so often misjudged efficiency of surgery, but as demonstration of an inaccurate diagnosis, based on a faulty physical examination.

In the same sense we must penetrate into the minutest details of leucorrhœa, laceration of the cervix, fistulæ, perimetritis, etc., etc.

To this point, more or less, I believe that every homœopathically thinking gynecologist will consent to my deductions; but I further believe that the same governing rule—as to this point observed—will hold its position for the question of major operations.

We must ask how far the vitality of a concrete case is endangered by the existing disease, to warrant an immediate interference with the knife; furthermore we must see to the nature and the danger of the disease, as in treating a certain disease—myoma—by drugs, with the reservation of a possible operation later on, I have seen results exactly as you are now witnessing the final steps in such a myoma cure.

Finally, I treat every case, and even those which only can be treated successfully with the knife—intraligamentous tumors—homœopathically, for the repeated experience that in every advance homœopathically treated case shows a vitality to resist shock, sepsis, etc. This point especially, if known to the homœopathic profession, I have never found deservedly mentioned. Again, in all cases where the imminent danger is proved, no time must be lost, as in large tumors, twisted pedicle, intra-abdominal hemorrhage, etc., etc.

Hahnemann in his *Organon* writes: "*The sensible physician will extract irritant particles causing inflammation of the cornea.*"

Here at this place I must most emphatically protest against certain teachings of the materia medica, that the homœopathic physician reads under *Ignatia* "ovarian hemorrhage, with sighing and sobbing, faintness, despondency."

I must remark to such a passus that it is impossible to diagnose ovarian hemorrhage. An ovarian hematoma—if not ovarian gestation—has *a priori* no symptom, and is therefore clinically in the sense of symptomatology of no consequence—and where symptoms should occur, so these symptoms will be those of a life-endangering hemorrhage; and here again the golden words of our Hahnemann of a century ago tell us what to do: "*The sensible physician will avert the danger of a hemorrhage by exposing and tying the wounded artery.*"

Here in this place I cannot refrain from making some remarks about the disease called appendicitis. I do not doubt that the immediate danger will be greatly checked by the indicated remedy; but how about the complications with salpingitis, etc.? How about the prognosis *quad vitam futuram?* "Crush a calculus, remove Belladonna berries" are again Hahnemann's words.

To sum up, I am following these rules:

1. That chronic cases, without endangered vitality, must be subjected to a fair trial of medicinal treatment.

2. That chronic cases must, by repeated physical examinations, be sharply classified for either medical or surgical treatment.

3. That every chronic disease rationally demanding a surgical interference, must be treated homœopathically, according to existing symptoms, before the operation.

4. That every suddenly-appearing danger to life must be treated according to the anatomical cause and origin.

With full deliberation I left the treatment of the malign growths until the present. My own observations in private practice and in my clinical work, and moreover the careful observation of the results of other gynecologists, have decided me to formulate the following rule:

Never to operate *a priori* on new growths recognized as malignant; the wish of the patient should be the only cause of interference. Just now the report of one of the foremost gynecologists of New York city is in my hands, showing his absolute negative results in curing malign growths. I have no doubt that this statement of Dr. Paul F. Mundé, whose books are in everybody's hands, will be fully believed, that all his extirpations for malign growths performed in his twelve years' service at Mt. Sinai Hospital were failures, that these patients suffered later from recidivus.

Ladies and gentlemen, I hope to have been able to show you the two-fold position of the homœopathic gynecologist at the present day, to discriminate between medicine and surgery, to be physician and surgeon in one person!

Let us again remember Hahnemann's words:

"*When the physician knows in each case the obstacles in the way of recovery, and how to remove them, he is prepared to act thoroughly and to the purpose, as a true master of the healing art.*"

Ladies and gentlemen, I now close my lecture regarding the rational treatment in gynecology; but it must be remembered that I owe all my results in the clinical practice, as also the foundation for my standpoint, in the full recognition of medicine besides surgery, to the teachings of Professor T. F. Allen from his *Organon* lectures.

